# A Bioinformatics Research on Novel Mechanism of Compound Kushen Injection for Treating Breast Cancer by Network Pharmacology and Molecular Docking Verification

**DOI:** 10.1155/2020/2758640

**Published:** 2020-08-10

**Authors:** Shuyu Liu, Xiaohong Hu, Xiaotian Fan, Ruiqi Jin, Wenqian Yang, Yifei Geng, Jiarui Wu

**Affiliations:** Department of Clinical Chinese Pharmacy, School of Chinese Materia Medica, Beijing University of Chinese Medicine, No. 11 North Three-Ring East Road, Chaoyang District, Beijing, China

## Abstract

Compound Kushen injection (CKI) has been extensively used in treating breast cancer (BC). However, the molecular mechanism remains unclear. In this study, 16 active compounds of CKI were obtained from 3 articles for target prediction. Then, a compound-predicted target network and a compound-BC target network were conducted by Cytoscape 3.6.1. The gene ontology (GO) enrichment analysis and Kyoto Encyclopedia of Genes and Genomes (KEGG) pathway enrichment analysis were performed on the DAVID database. The binding energy between the key targets of CKI and the active compounds was studied by molecular docking. As a result, 16 active compounds of CKI were identified, corresponding to 285 putative targets. The key targets of CKI for BC are HSD11B1, DPP4, MMP9, CDK1, MMP2, PTGS2, and CA14. The function enrichment analysis obtained 13 GO entries and 6 KEGG pathways, including bladder cancer, cancer pathways, chemical carcinogenesis, estrogen signaling pathway, TNF signaling pathway, and leukocyte transendothelial migration. The result of molecular docking indicated that DPP4 had strong binding activity with matrine, alicyclic protein, and sophoridine, and MMP9 had strong binding activity with adenine and sophoridine. In conclusion, the therapeutic effect of CKI on BC is based on the overall pharmacological effect formed by the combined effects of multiple components, multiple targets, and multiple pathways. This study provides a theoretical basis for further experimental research in the future.

## 1. Introduction

Although modern medicine has made great progress in cancer research, breast cancer (BC) remains an important health issue. BC is the most common cancer affecting women's health around the world, and its morbidity and mortality are expected to increase dramatically in the next few years [1]. Current clinical treatments for BC include surgical resection, chemoradiotherapy, and endocrine therapy, but these treatments will bring obvious side effects. Studies have shown that radiotherapy and chemotherapy can increase the risk of myelodysplastic disorder and acute myeloid leukemia in BC patients, which means more pain for patients with poor body function and poor tolerability [2]. Traditional Chinese medicine (TCM), as a traditional medicine for adjuvant treatment of tumors, has the effect of improving the immune function and tumor microenvironment of the patients and reducing the toxicity of radiotherapy and chemotherapy, so as to improve the survival rate [3–7]. It is shown that compound Kushen injection (CKI), as a TCM compound preparation, has a good synergistic antitumor effect by inhibiting tumor cell proliferation and inducing differentiation. It has been widely used in clinical practice in China [8]. CKI is prepared from a series of refined processes including percolation, boiling, and alcohol absorption of *Sophora flavescens* and *Smilax glabra*, which is able to clear heat and promote diuresis, cool blood and detoxify, resolve stagnation, as well as relieve pain [9]. The chemical composition of CKI is mainly composed of matrine alkaloids such as matrine and oxymatrine, which have significant antitumor activities [10]. *Sophora flavescens* alkaloids can play a role in regulating tumor cell proliferation, inducing tumor cell differentiation and apoptosis, and inhibiting tumor cell invasion and metastasis. It can also reduce tumor neovascularization and regulate body immunity [11]. At present, there are more and more clinical adjuvant treatment methods of CKI combined with radiotherapy and chemotherapy, which can improve the efficacy of radiotherapy or chemotherapy while reducing the resistance of chemotherapeutic drugs [12–18]. However, the mechanisms of CKI in treating BC remain unclear. With the rapid development of human society, the spectrum of human diseases has also undergone tremendous changes. The occurrence and development of complex diseases are closely related to multiple genes and signal pathways in the regulatory network of the body. It is difficult to achieve great therapeutic effects based on a single target. Network pharmacology is combined with high-throughput omics data analysis, computer virtual computing, and network database retrieval foundations. It not only embodies the new concept and mode of modern biomedical research but also changes the traditional mode of “one drug, one target, and one disease” in new drug development. Moreover, it has a profound impact on the concept, strategy, and method of certifying and discovering drugs [19]. Therefore, this study developed network pharmacology method, combined with molecular docking, to explore the anti-BC action mechanism of CKI primarily.

## 2. Materials and Methods

### 2.1. Active Ingredients and Potential Targets of CKI

There is a systematic and comprehensive search of published literature on the active compounds of CKI in CNKI, Wanfang, VIP database, and PubMed. The SMILES (Simplified Molecular Linear Input Specification) structures of the screened compounds were obtained using the PubChem database (https://pubchem.ncbi.nlm.nih.gov), and putative targets were collected from the Search Tool for Interacting Chemicals (STITCH, http://stitch.embl.de/), SuperPred (http://prediction.charite.de/), and Swiss Target Prediction (http://www.swisstargetprediction.ch/) databases and TCM Pharmacology Database and Analysis Platform (TCMSP, http://tcmspw.com/tcmsp.php) [20–23].

### 2.2. Collection of Target Proteins Related to BC

“Breast cancer” was identified as a keyword to collect proteins related to BC in the TTD databases (Therapeutic Target Database, http://db.idrblab.net/ttd/) [24]. At the same time, the Gene Expression Omnibus (GEO, https://www.ncbi.nlm.nih.gov/geo/) and The Cancer Genome Atlas (TCGA, http://cancergenome.nih.gov/) were applied to search differentially expressed genes of BC and then combined the obtained genes and removed duplicate data. The final results are the targets related to BC [25, 26].

### 2.3. Network Construction

The active compounds of CKI and the predicted targets were introduced into Cytoscape 3.6.1 to construct a compound-predicted target network. A compound-BC target network was set up by intersecting the predicted targets of the compounds with the targets related to BC. The network analyzer plug-in was used to analyze the key targets on the three key topological parameters of the network: degree, betweenness, and closeness. “Degree” refers to the number of connections between a node and other nodes in the network. “Betweenness” means the ratio of the shortest path through a node to the total number of paths through all nodes. “Closeness” shows the inverse of the sum of the distances of a node from other nodes [27]. The value of the above three topological parameters of a node is directly proportional to the importance of the node in the network.

### 2.4. Biological Functional and Pathway Enrichment Analysis

In this study, the DAVID (https://david.ncifcrf.gov/) platform was applied to perform GO functional enrichment analysis and KEGG pathway enrichment analysis on the potential key genes for anti-BC of the CKI obtained after topology analysis [28]. GO is a database that annotates genes and protein functions into three main items: cellular components (CCs), molecular functions (MFs), and biological processes (BPs). Pathway enrichment analysis revealed possible biological processes for key genes [29, 30]. The results of the enrichment analysis are visualized by the GO plot package in R software.

### 2.5. Molecular Docking

The SDF format files of compounds were downloaded from the PubChem database and converted into mol2 format files. Protein conformation screening on key targets in the RCSB PDB database (https://www.rcsb.org/) was performed, and the PDB format files were downloaded [31]. The following are the screening conditions: (1) the protein structure is obtained by X-crystal diffraction; (2) the resolution of the protein crystal is less than 3 Å; (3) the protein structure reported by molecular docking is preferred; and (4) the biological source of the protein structure is human beings. Water molecules and original small-molecule ligands were deleted from the protein structure. The position of the active pocket was determined after performing hydrogenation, giving charge, and combining nonpolar hydrogen using the AutoDockTools 1.5.6 software. At the same time, grid box coordinates and box size were established. Finally, AutoDock Vina 1.1.2 was used to perform docking operations [32]. The receptor-ligand pairs were sorted and screened according to the affinity (kcal/mol). Ultimately, PyMOL 2.3.2 software was used for visual processing to check the binding status of ligands and receptor binding sites [33].

## 3. Results

### 3.1. Compound-Predicted Target Network

**Figure 1:**
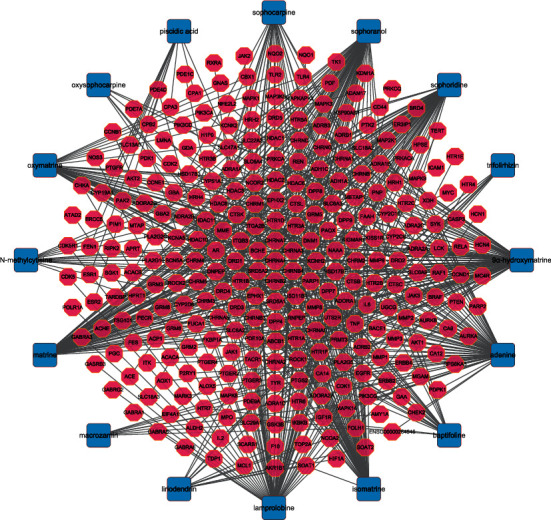
Compound-target network. Notes: the blue squares represent active compounds of CKI, and the pink octagons represent the targets of these compounds.

As shown in Table 1, a total of 16 active compounds in CKI and 285 predicted targets were obtained [34–36]. The compound-predicted target network (Figure 1) includes 301 nodes (16 compounds and 285 targets) and 636 edges. Topological analysis illustrated that most targets are simultaneously regulated by multiple chemical components, such as neuronal acetylcholine (NA) receptor subunit target (CHRNA4, CHRNB2, CHRNA3, CHRNB4, CHRNA7), 3-oxo-5-alpha-steroid 4-dehydrogenase 1 (SRD5A1), cholinesterase, BCHE, and corticosteroid 11-beta-dehydrogenase isozyme 1 (HSD11B1). 9*α*-hydroxymatrine, lamprolobine, adenine, matrine, sophoranol, sophoridine, and isomatrine have a degree over 50, indicating that the active compounds of CKI play a role in the treatment of breast cancer by regulating multiple targets, which conforms to the TCM characteristics of multicomponent, multitarget, and multidisease (Supplementary Table 1).

### 3.2. Compound-BC Target Network Diagram

**Figure 2:**
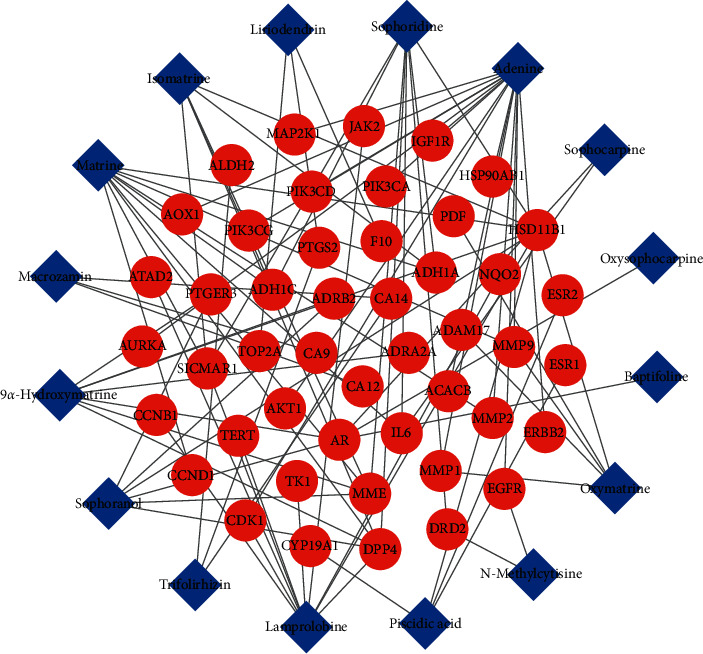
Compound-BC target network. Notes: the blue diamonds represent active compounds of CKI, and the red circles represent the common targets.

The compound-BC target network was constructed with the obtained 47 common targets and 16 corresponding compounds (Figure 2). The key nodes with high connectivity in the network can be distinguished from other nodes through node topology analysis. Therefore, the nodes having the degree, betweenness, and closeness greater than their corresponding median values (degrees ≥ 1.85, betweenness ≥ 0.04, and closeness value ≥ 0.29) are selected as the key nodes of the network. Finally, 7 key targets are obtained, namely, corticosteroid 11-beta-dehydrogenase isozyme 1 (HSD11B1), dipeptidyl peptidase 4 (DPP4), matrix metalloproteinase-9 (MMP9), cyclin-dependent kinase 1 (CDK1), 72 kDa type-IV collagenase (MMP2), prostaglandin G/H synthase 2 (PTGS2), and carbonic anhydrase 14 (CA14). It is predicted that these seven targets may play a key role in the treatment of BC.

### 3.3. GO and KEGG Pathway Enrichment Analysis

**Figure 3:**
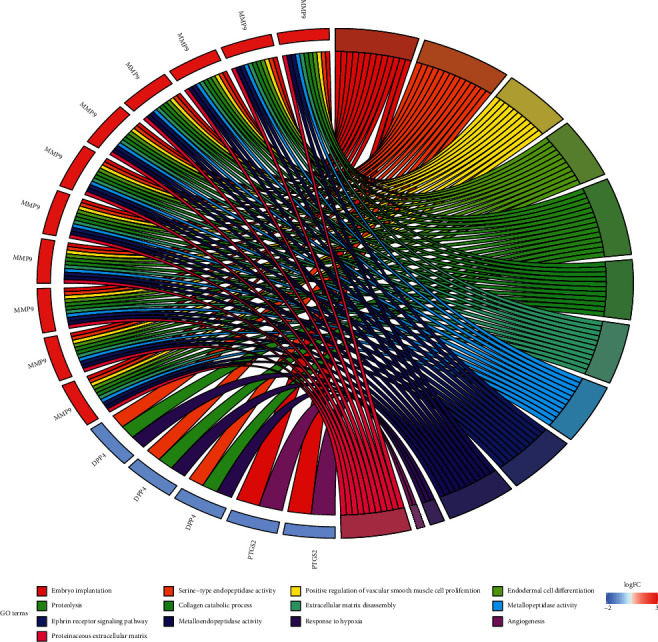
GO functional enrichment analysis of key targets.

To further explore biological processes, molecular functions, and signaling pathways, GO functional enrichment analysis and KEGG pathway enrichment analysis were performed for 7 key targets from the compound-BC target network. 13 enriched GO items were obtained finally. Items related to biological processes (BP) were embryo implantation (GO: 0007566), positive regulation of vascular smooth muscle cell proliferation (GO: 1904707), endodermal cell differentiation (GO: 0035987), proteolysis (GO: 0006508), collagen catabolic progress (GO: 0030574), extracellular matrix disassembly (GO: 0022617), ephrin receptor signaling pathway (GO: 0048013), response to hypoxia (GO: 0001666), and angiogenesis (GO: 0001525); items related to molecular function (MF) include serine-type endopeptidase activity (GO: 0004252), metallopeptidase activity (GO: 0008237), and metalloendopeptidase activity (GO: 0004222) related to MF; and the GO entry related to cell composition (CC) is proteinaceous extracellular matrix (GO: 0005578) (Figure 3). It suggests that the active compounds of CKI may exert anti-BC effects by participating in various biological regulation processes.

**Figure 4:**
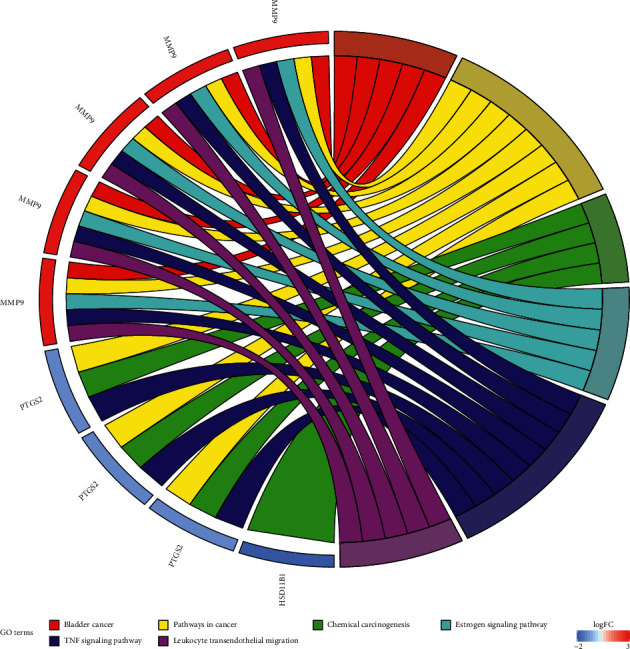
KEGG pathway enrichment analysis of key targets.

**Figure 5:**
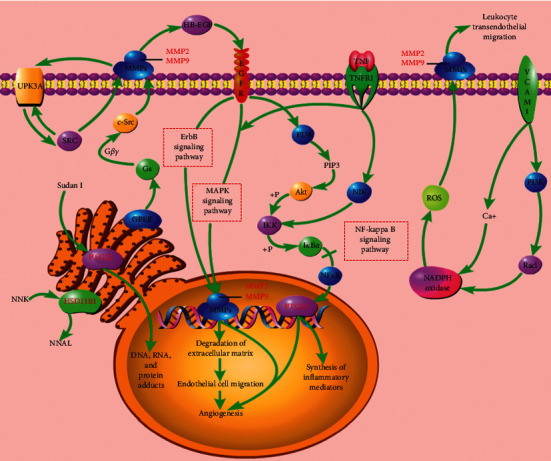
Illustration of crucial biological progress caused by key targets and known therapeutic targets for CKI.

**Figure 6:**
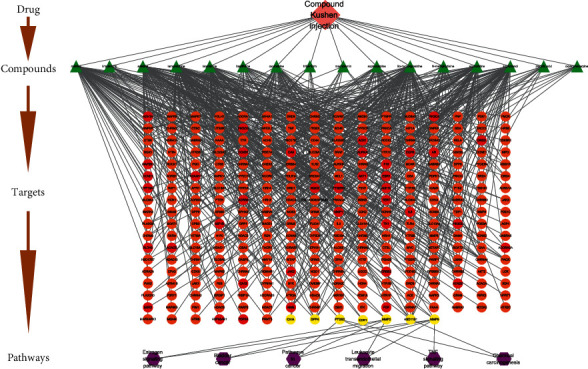
Compound-target-pathway network. Notes: The pink diamond refers to CKI. The green triangles represent active compounds in CKI. The orange, red, and yellow circles represent putative targets of CKI. Among them, the red circles represent the common targets of CKI and BC, and the yellow circles represent key targets of CKI for the treatment of BC. The purple hexagons represent the main pathways of key targets.

KEGG enrichment analysis totally obtained 6 pathways, namely, bladder cancer (hsa05219), pathways in cancer (hsa05200), chemical carcinogenesis (hsa05204), estrogen signaling pathway (hsa04915), TNF signaling pathway (hsa04668), and leukocyte transendothelial migration (hsa04670) (Figure 4). These pathways involve cancer, endocrine system, signaling system, and immune system.  Figure 5 shows the main biological effects of the key targets of CKI, in addition to the major enriched pathways mentioned above; the whole biological process also involves ErbB signaling pathway, MAPK signaling pathway, and NF-kappa B signaling pathway.  Figure 6 illustrates the interaction among compounds, targets, and pathways of CKI in treating BC.

### 3.4. Molecular Docking

**Figure 7:**
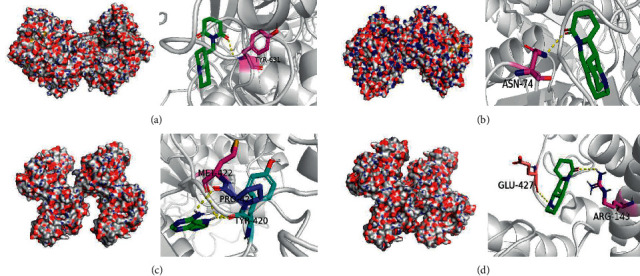
Result of molecular docking. Notes: (a) lamprolobine acts on DPP4; (b) sophoridine acts on DPP4; (c) adenine acts on MMP9; (d) sophoridine acts on MMP9.

The structure of key targets and compound with highest value were introduced into AutoDockTools 1.5.6 for molecular docking (Tables 2 and 3). In general, the lower the binding free energy, the more stable the binding between the ligand and protein receptor. According to the results of molecular docking, the macromolecular protein receptor DPP4 has strong binding activity with matrine, lamprolobine, and sophoridine, and MMP9 has strong binding activity with adenine and sophoridine. The hydrogen-bonding relationship between the active small-molecule ligands and the protein receptor is shown in Figure 7.

## 4. Discussion

BC is a common malignant tumor that threatens women's health and lives. In clinical practice, patients are often treated with integration of TCM and western medicine. Although the effect of traditional Chinese medicine on tumor shrinkage is not as obvious as that of radiotherapy and chemotherapy, it has less toxic and side effects and high safety, which helps slow down the clinical condition and improve the quality of life of patients [37]. A large number of studies have shown that CKI has a good intervention effect against the toxicity of chemotherapeutic drugs, which can enhance the immune function and protect the hematopoietic system.

In this study, the interpretation of the mechanism of CKI in the treatment of BC was performed by integrated target prediction, network construction, and molecular docking. First, 16 active compounds were found in CKI, and 285 potential targets were obtained by target prediction. It is reported that matrine alkaloids have analgesic, heart-strengthening, antiarrhythmic, antiviral, anti-inflammatory, antitumor, swelling and diuretic, immunosuppressive, antibacterial, and insecticidal effects [38]. Matrine has a wide range of biological activities, such as antibacterial, antiviral, anti-inflammatory, immunomodulatory, anti-tumor, and has positive muscle strength, negative frequency, and antiarrhythmic to the heart [39]. Studies have shown that matrine has antitumor effects, can inhibit the growth of BC-MDA-MB-231 and MCF-7 cells, and induce their apoptosis. It may be a new BC inhibitor [40, 41]. Sophoridine is also an alkaloid with significant antitumor effect. It can directly kill tumor cells and exerts its antitumor effect by impacting the cell cycle [42].

Second, it was found that HSD11B1, DPP4, MMP9, CDK1, MMP2, PTGS2, and CA14 may be key targets through a comprehensive analysis of the compound-predicted target network and compound-BC target network. These targets participate in the regulation of cell cycle, cell proliferation, inflammation induction, and other related proteins. Heather-SF conducted a nested case-control study on the HSD11B1 gene region and found that the HSD11B1 gene region may contain BC risk alleles, and the common variation may be related to BC in postmenopausal white women [43]. MMP2 and MMP9 were found to be involved in the invasion and metastasis of BC, and their levels were significantly increased in the serum and plasma of BC patients [44]. Studies have shown that the expression of MMP2 and MMP9 in BC may be related to the expression of AP-2 and HER2. The positive expression of MMP9 indicates the low survival rate of small hormone reactive tumors, and its expression in cancer cells is beneficial to the survival of tumors [45]. CDK1 is a protein-encoding gene of the cell-cycle-dependent kinase family and also plays an important regulatory role controlling cell cycle. CDK disorder leads to the increase of cell proliferation, which has been found in many cancers including breast cancer. Studies have shown that many genes can negatively regulate the expression of CDK1 mRNA by selectively blocking CDK1 or in combination with other therapies, thus inhibiting the proliferation of human BC cells and blocking G2/M cells [46–48]. The application is related to anticancer effects, which suggests that CDK1 may be considered to be the best CDK target for BC treatment [49]. PTGS2, also known as cyclooxygenase (COX)-2, is an inflammation-inducing enzyme. Studies have shown that PTGS2 is upregulated in approximately 40% of BC patients (including ductal carcinoma in situ and invasive cancer) and associated with metastasis diseases, which reduced patients' survival rate [50].

According to the GO functional enrichment analysis and KEGG pathway enrichment analysis results, the key targets of the active compound regulation of CKI are significantly enriched in a variety of biological processes, molecular functions, and cellular components, such as embryo implantation, positive regulation of vascular smooth muscle cell proliferation, endoderm cell differentiation, proteolysis, collagen catabolism, extracellular matrix breakdown, adrenergic receptor signaling pathway, hypoxia, angiogenesis, serine-type endopeptidase activity, metallopeptidase activity, metalloendopeptidase activity, and protein extracellular matrix. The main enriched pathways are cancer pathway, chemical carcinogenesis, estrogen signaling pathway, TNF signaling pathway, and leukocyte transendothelial migration. Chemical carcinogenesis refers to the characteristics of some cancer cells, such as nongenotoxic carcinogens exposed to the environment, which changes the signal transduction pathway and eventually leads to high variability, genomic instability, uncontrolled proliferation, and resistance to apoptosis [51]. The estrogen signaling pathway enables estrogen to bind to the estrogen receptors ER*α* and ER*β*. It plays an opposite role in cell proliferation, apoptosis, and migration and has different effects on the occurrence and development of tumor by inducing different transcription reactions [52]. Tumor necrosis factor (TNF), as an important cytokine, plays an important role in many physiological and pathological processes such as cell proliferation, differentiation, apoptosis, immune regulation, and inflammation induction [53]. Studies have suggested that TNF-*α* sign plays a key role in BC cell migration and its level has great potential to be prognostic cancer biomarkers [54]. The migration of white blood cells from the blood into tissues is essential for immune surveillance and inflammation. During inflammation or immune surveillance, leukocytes in the blood pass through endothelial cells in the vascular lumen and migrate to the next layer of tissue. This process is called leukocyte transendothelial cell migration (TEM). The mechanism of CKI against BC may be closely related to the regulation of BC cell proliferation, apoptosis and migration, immune regulation, and inflammation induction.

## 5. Conclusion

In summary, this study revealed the potential pharmacological mechanism of CKI in the treatment of BC at a system level, which may involve synergistic regulation of cell proliferation, apoptosis, cell migration, immune regulation, and inflammation induction. Besides, the present study provides clues to understand and evaluate the synergistic effect of TCM in the treatment of complex diseases. Considering that this research is mainly based on data analysis, further biological experiments are essential for verifying the result.

## Figures and Tables

**Table 1:** Active compounds of CKI.

Compounds	PubChem CID	MW (g/mol)	Structure
9*α*-Hydroxymatrine	15385684	264.369	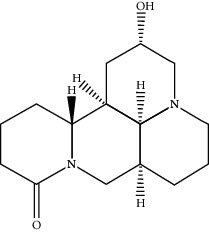
Adenine	190	135.13	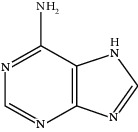
Baptifoline	621307	260.337	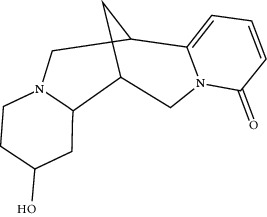
Isomatrine	5271984	248.37	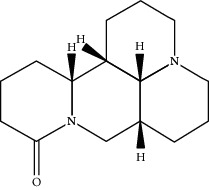
Lamprolobine	87752	264.369	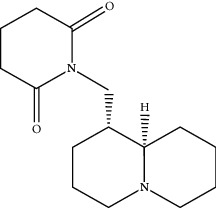
Liriodendrin	21603207	742.724	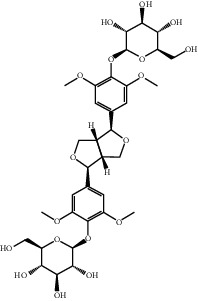
Macrozamin	9576780	384.338	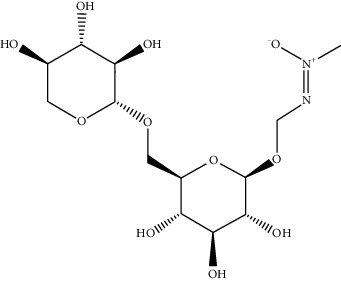
Matrine	91466	248.37	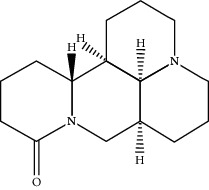
N-Methylcytisine	670971	204.273	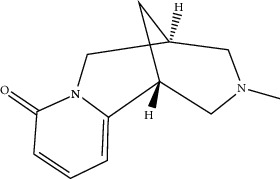
Oxymatrine	114850	264.369	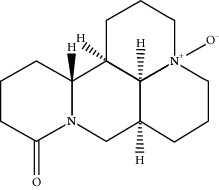
Oxysophocarpine	24721085	262.353	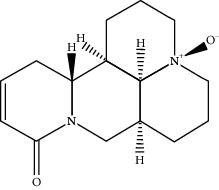
Piscidic acid	6710641	256.21	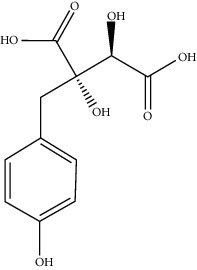
Sophocarpine	115269	246.354	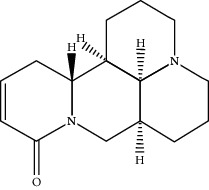
Sophoranol	12442899	264.369	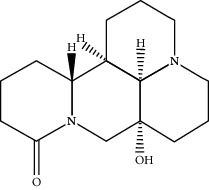
Sophoridine	165549	248.37	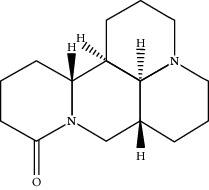
Trifolirhizin	442827	446.408	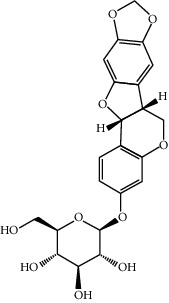

**Table 2:** The information of key targets.

Protein name	Target	PDB ID
Corticosteroid 11-beta-dehydrogenase isozyme 1	HSD11B1	2ILT
Dipeptidyl peptidase 4	DPP4	4A5S
Matrix metalloproteinase-9	MMP9	5TH6
Cyclin-dependent kinase 1	CDK1	4YC3
72 kDa type-IV collagenase	MMP2	4WKE
Prostaglandin G/H synthase 2	PTGS2	5KIR
Carbonic anhydrase 14	CA14	4LU3

**Table 3:** The docking information.

Compound	Affinity (kal·mol^−1^)		
	HSD11B1	DPP4	MMP9
Lamprolobine	−5.4	−7.3	−6.1
Sophoridine	−6.2	−7.9	−7.2
Matrine	−6	−7.4	−6.1
Adenine	−6.1	−5.8	−7

## Data Availability

The data used to support the findings of this study are available from the corresponding author upon request.

## Abbreviations

CKI: Compound Kushen injection
BC: Breast cancer
TCM: Traditional Chinese medicine
GO: Genome ontology
KEGG: Kyoto Encyclopedia of Genes and Genomes
DAVID: Database for Annotation, Visualization, and Integrated Discovery
SMILES: Simplified Molecular Linear Input Specification
STITCH: Search Tool for Interacting Chemicals
TCMSP: Traditional Chinese Medicine Systems Pharmacology Analysis Platform
TTD: Therapeutic Target Database
GEO: Gene Expression Omnibus
TCGA: The Cancer Genome Atlas
CCs: Cellular components
MFs: Molecular functions
BPs: Biological processes
TNF: Tumor necrosis factor
TEM: Transendothelial cell migration
NA: Neuronal acetylcholine
SRD5A1: 3-Oxo-5-alpha-steroid 4-dehydrogenase 1
HSD11B1: Corticosteroid 11-beta-dehydrogenase isozyme 1
DPP4: Dipeptidyl peptidase 4
MMP9: Matrix metalloproteinase-9
CDK1: Cyclin-dependent kinase 1
MMP2: 72 kDa type-IV collagenase
PTGS2: Prostaglandin G/H synthase 2
CA14: Carbonic anhydrase 14
MMPs: Matrix metalloproteinases.

## Supplementary Materials

Supplementary Materials Supplementary Table 1: Degree of compounds in compound-compound target network. Supplementary Table 2: the topological analysis of key targets. Supplementary Table 3: the result of GO enrichment analysis. Supplementary Table 4: the result of KEGG pathway enrichment.
