# Supplementary material for: A Bioinformatics Research on Novel Mechanism of Compound Kushen Injection for Treating Breast Cancer by Network Pharmacology and Molecular Docking Verification
Source: Evid Based Complement Alternat Med. 2020 Aug 10;2020:2758640. doi: 10.1155/2020/2758640 (PMC7439201; doi:10.1155/2020/2758640)
Supplement: Supplementary Materials — Supplementary Table 1: Degree of compounds in compound-compound target network. Supplementary Table 2: the topological analysis of key targets. Supplementary Table 3: the result of GO enrichment analysis. Supplementary Table 4: the result of KEGG pathway enrichment. [file 2758640.f1.pdf]

Suppelymentary table 1: degree of compounds in Compound-compound target network.

Compound	Degree
9α-hydroxymatrine	85
lamprolobine	83
adenine	71
matrine	67
sophoranol	62
sophoridine	59
isomatrine	54
oxymatrine	40
sophocarpine	29
baptifoline	24
N-methylcytisine	15
liriodendrin	14
piscidic acid	11
macrozamin	8
trifolirhizin	8
oxysophocarpine	6

Supplementary table 2: The topological analysis of key targets.

Target	Degree	Betweenness	Closeness
HSD11B1	8	0.333954	0.416
DPP4	6	0.098328	0.368794
MMP9	3	0.12431	0.363636
CDK1	2	0.109152	0.348993
MMP2	2	0.081926	0.348993
PTGS2	2	0.5	0.428571
CA14	2	0.5	0.428571
median values	1.851064	0.043259	0.292373

Supplementary table 3: The result of GO enrichment analysis.

Category	ID	Term	Genes	adj_pvalue
BP	GO:0007566	embryo implantation	PTGS2, MMP9, MMP2	0.1089
MF	GO:0004252	serine-type endopeptidase activity	MMP9, MMP2, DPP4	2.823119
BP	GO:1904707	positive regulation of vascular smooth muscle cell proliferation	MMP9, MMP2	6.217181
BP	GO:0035987	endodermal cell differentiation	MMP9, MMP2	10.91513
BP	GO:0006508	proteolysis	MMP9, MMP2, DPP4	13.72433
BP	GO:0030574	collagen catabolic process	MMP9, MMP2	23.98745
BP	GO:0022617	extracellular matrix disassembly	MMP9, MMP2	27.80609
MF	GO:0008237	metallopeptidase activity	MMP9, MMP2	22.2704
BP	GO:0048013	ephrin receptor signaling pathway	MMP9, MMP2	30.84329
MF	GO:0004222	metalloendopeptidase activity	MMP9, MMP2	29.65782
BP	GO:0001666	response to hypoxia	MMP2, DPP4	52.26445
BP	GO:0001525	angiogenesis	PTGS2, MMP2	61.71967
CC	GO:0005578	proteinaceous extracellular matrix	MMP9, MMP2	54.74916

Supplementary table 4: The result of KEGG pathway enrichment.

Term	Genes	FDR
hsa05219: Bladder cancer	MMP9, MMP2	26.8888
hsa05200: Pathways in cancer	PTGS2, MMP9, MMP2	31.17162
hsa05204: Chemical carcinogenesis	PTGS2, HSD11B1	45.81944
hsa04915: Estrogen signaling pathway	MMP9, MMP2	53.20807
hsa04668: TNF signaling pathway	PTGS2, MMP9	56.01464
hsa04670: Leukocyte transendothelial migration	MMP9, MMP2	58.65591
